# Evaluation of synthetic formaldehyde and methanol assimilation pathways in *Yarrowia lipolytica*

**DOI:** 10.1186/s40694-019-0090-9

**Published:** 2019-12-17

**Authors:** Eija Vartiainen, Peter Blomberg, Marja Ilmén, Martina Andberg, Mervi Toivari, Merja Penttilä

**Affiliations:** VTT Technical Research Centre of Finland Ltd, P.O. Box 1000, 02044 VTT Espoo, Finland

**Keywords:** *Yarrowia lipolytica*, Crude glycerol, Methanol, Formaldehyde

## Abstract

**Background:**

Crude glycerol coming from biodiesel production is an attractive carbon source for biological production of chemicals. The major impurity in preparations of crude glycerol is methanol, which is toxic for most microbes. Development of microbes, which would not only tolerate the methanol, but also use it as co-substrate, would increase the feasibility of bioprocesses using crude glycerol as substrate.

**Results:**

To prevent methanol conversion to CO_2_ via formaldehyde and formate, the formaldehyde dehydrogenase (FLD) gene was identified in and deleted from *Yarrowia lipolytica.* The deletion strain was able to convert methanol to formaldehyde without expression of heterologous methanol dehydrogenases. Further, it was shown that expression of heterologous formaldehyde assimilating enzymes could complement the deletion of FLD. The expression of either 3-hexulose-6-phosphate synthase (HPS) enzyme of ribulose monosphosphate pathway or dihydroxyacetone synthase (DHAS) enzyme of xylulose monosphosphate pathway restored the formaldehyde tolerance of the formaldehyde sensitive Δ*fld1* strain.

**Conclusions:**

In silico, the expression of heterologous formaldehyde assimilation pathways enable *Y. lipolytica* to use methanol as substrate for growth and metabolite production. In vivo, methanol was shown to be converted to formaldehyde and the enzymes of formaldehyde assimilation were actively expressed in this yeast. However, further development is required to enable *Y. lipolytica* to efficiently use methanol as co-substrate with glycerol.

## Background

Yeast *Yarrowia lipolytica* is a non-pathogenic obligate aerobe which has potential for many industrial applications, such as production of organic acids, aroma compounds, enzymes and lipids [1, 2]. *Y. lipolytica* is or has been used in commercial processes for production of, e.g. single cell proteins (SCP), citric acid, erythritol, omega-3-eicosapentaenoic acid (EPA) and lysosomal enzymes [3, 4].

Recently, *Y. lipolytica* has come up as an appealing host for applications which use crude glycerol as a carbon source [5, 6]. Crude glycerol is a by-product of biodiesel production, in which lipids are chemically transesterified using small alcohols, such as methanol or ethanol [7, 8]. In the process, glycerol is produced at the level of roughly 10 wt% of the main product biodiesel (fatty acid methyl esters). Depending on the details of the process, different levels of other by-products such as alcohols, salts, heavy metals and water are present in the glycerol fraction [9].

For chemical conversion of crude glycerol, purification of the substrate is required. Bioconversion allows the use of crude glycerol without further purification [10–12]. Oleaginous yeasts can be used to produce microbial oils from crude glycerol, thus enhancing the feasibility of the biodiesel process [10]. In addition, these yeasts could be used for the conversion of crude glycerol to higher value chemicals such as different fatty acids or biopolymer components [13, 14].

Besides water, the major impurity in crude glycerol is methanol, which is known to have inhibitory effects on microbial growth [15, 16]. The methanol concentration varies considerably from batch to batch, but it can be relatively high. In the study by Thompson and He [17], methanol contents up to 37.5 (wt)% were found in crude glycerol. Although methanol can be removed by evaporation, it requires energy and thus the process would be more efficient if the micro-organism tolerated the methanol. Recently, Iyyappan et al. used an adaptation strategy to increase the methanol tolerance of an *Aspergillus niger* strain for production of malic acid from crude glycerol [18]. In the most desirable process, however, the organism would not only tolerate methanol, but also use it as a co-substrate with glycerol.

Native methanol-utilising microbes oxidise methanol to formaldehyde, which can either be dissimilated to carbon dioxide or assimilated to biomass. Methanol oxidation is catalysed by methanol dehydrogenases, which are divided to three classes based on their electron acceptors (reviewed by [19]). Prokaryotes use either NAD or PQQ-dependent methanol dehydrogenases while eukaryotic (yeast) methanol dehydrogenases use oxygen as the electron acceptor. The NAD dependent methanol dehydrogenases (EC 1.1.1.244) of gram positive methylotrophic bacteria have been successfully expressed in *Corynebacterium glutamicum* and *Escherichia coli* [20, 21]. In addition, the alcohol oxidase (EC 1.1.3.13) from yeast *Pichia pastoris* was recently introduced to *Saccharomyces cerevisiae* [22].

Methanol oxidation leads to formaldehyde production inside cells. Formaldehyde, which is produced also in other cellular processes, is highly cytotoxic and thus all organisms possess formaldehyde detoxification pathways [23]. The detoxification of formaldehyde can happen enzymatically, but a common mechanism is a non-enzymatic binding of formaldehyde to thiol or pterin [23, 24]. After the non-enzymatic step, formaldehyde is further converted by enzymatic mechanisms. In yeasts (both methylotrophic and non-methylotrophic), formaldehyde is detoxified through a glutathione-dependent pathway. Glutathione-bound formaldehyde is oxidised to carbon dioxide by the actions of formaldehyde dehydrogenase (FLD; EC 1.2.1.1) and formate dehydrogenase (FDH; EC 1.2.1.2) [25–27].

In methylotrophic organisms, there exists formaldehyde assimilation pathways in addition to formaldehyde dissimilation pathways. In methylotrophic prokaryotes, the conversion of formaldehyde to biomass components occur either through the serine cycle or the ribulose monophosphate pathway (RuMP) [28, 29]. In the serine cycle of alpha-proteobacteria, formaldehyde is converted to methylene tetrahydrofolate either enzymatically or via a non-enzymatic reaction. Methylene tetrahydrofolate condenses with glycine to form serine. Glycine is then regenerated through a series of enzymatic reactions thus closing the cycle (reviewed by [30]). The RuMP pathway for formaldehyde assimilation is widespread in prokaryotes [29]. In the first step of the pathway, formaldehyde is condensed with ribulose-5-phosphate, a reaction catalysed by 3-hexulose-6-phosphate synthase (HPS, (EC 4.1.2.43) [31]. The resulting 3-hexulose-6-phosphate is then converted to fructose-6-phosphate by 6-phospho-3-hexuloisomerase (PHI, EC 5.3.1.27) [32].

Methylotrophic eukaryotes utilise the xylulose monophosphate pathway (XuMP) for formaldehyde assimilation [33]. The enzymes of the XuMP pathway are located in peroxisomes along with the methanol oxidising enzyme [34]. In the XuMP pathway, dihydroxyacetone synthase (formaldehyde transketolase, EC 2.2.1.3) condenses formaldehyde with xylulose-5-phosphate forming dihydroxyacetone and glyceraldehyde-3-phosphate. In both RuMP and XuMP pathways, the pentose monophosphates are regenerated through transaldolases and transketolases in a manner similar to the Calvin cycle and the pentose phosphate pathway [29, 34].

The aim of this study was to explore the methanol and formaldehyde assimilation pathways in the oleaginous yeast *Y. lipolytica* to enable the efficient use of crude glycerol as a substrate for fermentation processes. First, the whole pathway from methanol to biomass was assessed by metabolic modelling. Then, formaldehyde assimilation pathways of both prokaryotic and eukaryotic origin were introduced into *Y. lipolytica* and their functionality in this yeast was studied. In addition, a sequence search was conducted for finding active HPS enzymes for formaldehyde assimilation in eukaryotic hosts.

## Results

### Selection of metabolic pathways for methanol utilisation in *Yarrowia lipolytica*

Methanol utilisation requires two steps: first, the methanol is converted to formaldehyde and second, formaldehyde is assimilated to central carbon metabolism. We reasoned that the first step could be performed by native alcohol dehydrogenases present in *Y. lipolytica*. This reaction, which utilises NADH as a co-factor, is highly temperature-dependent. We thus calculated the reaction thermodynamics in conditions which *Y. lipolytica* can grow.

The calculation of the Gibbs energy of reaction at temperatures other than the standard temperature 25 °C requires the enthalpy, the entropy, and the temperature-dependent heat capacity of reaction. These thermodynamic parameters are typically obtained from experimental data. However, the literature data for formaldehyde is inconsistent due to the experimental difficulties arising from the spontaneous hydration of formaldehyde to methylene glycol [35]. This prompted a thorough analysis and reconciliation of the available thermodynamic data (see Additional file 1: Document S1). The reconciled value for the standard Gibbs energy of formation for the equilibrium mixture of formaldehyde and methylene glycol (i.e. partially-hydrolysed formaldehyde) is concluded to be 130.54 kJ/mol ± 3.3 kJ/mol (see Additional file 1: Document S1 for details). The equilibrium concentration of partially-hydrolysed formaldehyde, in the alcohol dehydrogenase reaction medium having 0.5 M methanol, was estimated at 28 °C, 30 °C, and 50 °C based on the reconciled data to be 34, 40, and 170 µM, respectively.

**Fig. 1:**
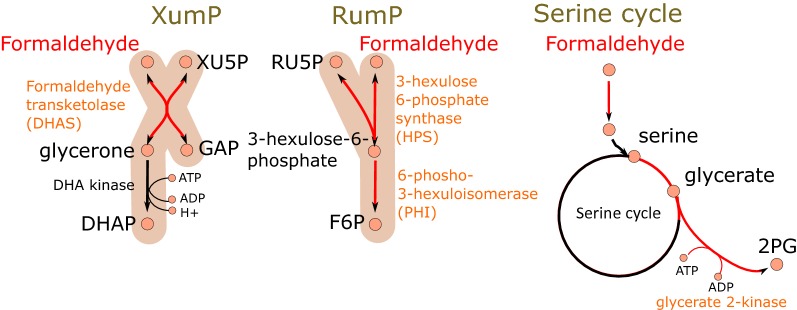
Formaldehyde assimilation pathways

To compare the feasibility of heterologous formaldehyde assimilation pathways, three different pathways, namely RuMP, XuMP and Serine cycle, for metabolism of formaldehyde were separately added to the genome scale metabolic model iNL895 for *Y.* *lipolytica* (Fig. 1, Additional file 2: Document S2). In addition, alcohol dehydrogenase reaction (r_0181) was allowed to make the conversion between methanol and formaldehyde. The model was optimised for growth or for triacylglycerol production while methanol (6 cmol/CDW/h) was allowed as the only carbon source. The results showed that all three formaldehyde assimilation pathways allowed the model to use methanol as a sole carbon source for growth and for TAG production (Table 1).

**Table 1:** Calculated in silico growth rate and yield for triacylglycerol (TAG) with methanol (6 C-mol g DW^−1^ h^−1^) used as substrate and the heterologous pathways RuMP, XuMP and Serine cycle added to the model

	Optimised for growth		Optimised for triacylglycerol (TAG)	
	Growth rate	Oxygen demand	Yield	Oxygen demand
	(1/h)	(mmol/cmol methanol)	(mol/mol methanol)	(mmol/cmol methanol)
RuMP	0.13	0.74	0.50	0.74
XuMP	0.11	0.89	0.50	0.74
Serine cycle	0.08	1.02	0.49	0.76

### Methanol tolerance of the wild type *Yarrowia lipolytica* strain

**Fig. 2:**
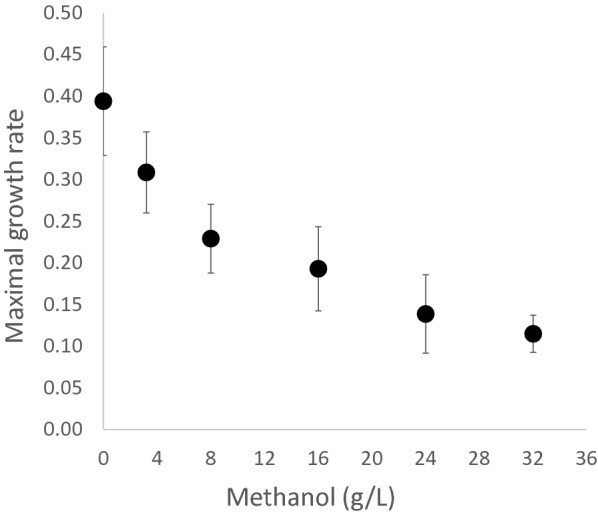
Methanol tolerance of wild type *Yarrowia lipolytica* VTT C-00365. The data is derived from three independent experiments

The effect of methanol on the growth of wild type *Y. lipolytica* VTT C-00365 was tested in microtiter plate scale-experiments. Cells were grown on 0.2 M glycerol in the presence of different concentrations of methanol (0, 0.1, 0.25, 0.5, 0.75 and 1 M) (Fig. 2). Under these conditions, addition of 0.1 M or more methanol reduced the maximal growth rate on glycerol statistically significantly (two-way anova, p < 0.01). Addition of 1 M methanol led to growth rate which was approximately 33% of the maximal growth rate without methanol. We also tested the widely used *Y. lipolytica* W29 strain, but the growth on glycerol was affected already on 0.1 M methanol to the extent that the growth rates could not be reliably calculated (data not shown).

### Identification and deletion of formaldehyde dehydrogenase gene

**Fig. 3:**
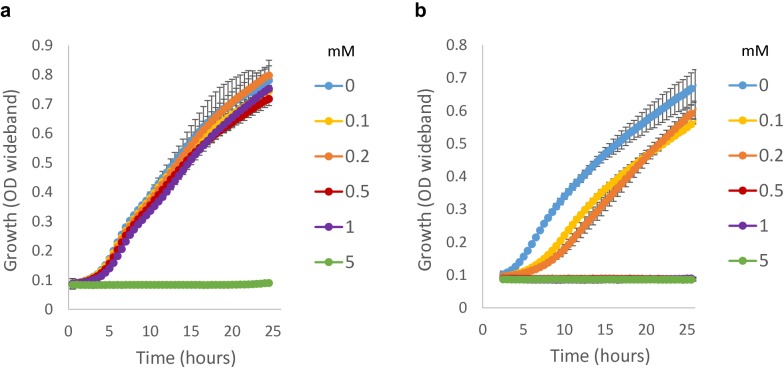
Formaldehyde tolerance of wild type (**a**) and Δ*fld1* (**b**) strains during growth on 2% glycerol. In **b** 0.5 mM, 1 mM lines are under 5 mM line

To prevent formaldehyde dissimilation, the formaldehyde dehydrogenase gene was deleted from strain VTT C-00365. The formaldehyde dehydrogenase gene of *Y. lipolytica* was not previously known and was identified based on homology to *S. cerevisiae* formaldehyde dehydrogenase SFA1. The protein predicted to be encoded by open reading frame YaliOF09603 had 70% identity with SFA1 and was the only candidate for a formaldehyde dehydrogenase gene of *Y. lipolytca*. YaliOF09603 was named *fld1* and deleted from VTTC–00365 strain to generate the formaldehyde dehydrogenase deletion strain Δ*fld1*. The growth of this strain on 0.2 M glycerol was affected by the addition of 0.1–0.2 mM formaldehyde and completely abolished by addition of 0.5 mM formaldehyde. The growth of wild type strain was only affected by formaldehyde concentration above 1 mM (Fig. 3). In addition, the deletion of FLD led to reduced growth even without the supplementation of formaldehyde in the growth medium.

### Conversion of methanol to formaldehyde

**Fig. 4:**
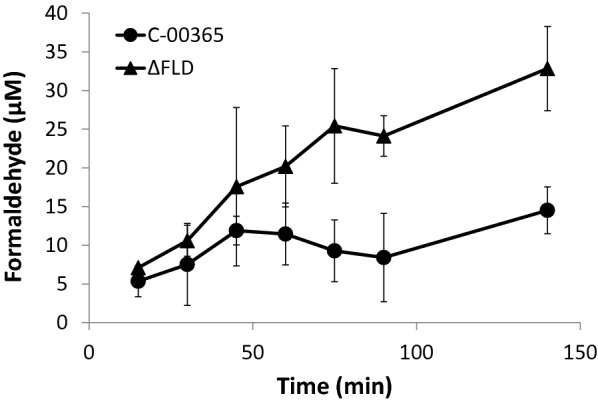
Extracellular formaldehyde concentration after addition of 0.5 M methanol

To address the first step in the methanol utilisation pathway, the ability of *Y. lipolytica* VTT C-00365 to produce formaldehyde from methanol with endogenous alcohol dehydrogenases was tested. For assessing the production of formaldehyde from methanol, wild type and Δ*fld1* strains were incubated at + 28 °C in minimal medium containing 0.5 M methanol as the only carbon source (Fig. 4). After 140 min, a formaldehyde concentration of 30 µM was detected with the formaldehyde dehydrogenase deletion strain.

Formaldehyde production from methanol is thought to be due to the activity of alcohol dehydrogenases. The measured alcohol dehydrogenase activity with methanol as substrate was 0.4–1 mU/mg total soluble protein in wild type *Y. lipolytica* cells while with ethanol as substrate, the activity was 40–80 mU/mg total soluble protein.

### Formaldehyde tolerance of the strains expressing HPS enzyme of ribulose monophosphate pathway or DHAS enzyme of xylulose monophosphate pathway

Formaldehyde conversion by enzymes of either RumP or XuMP pathways was tested by expressing these enzymes in a centromeric plasmid in the Δ*fld1* strain. To avoid the possibility of native, peroxisomal, localisation of DHAS enzyme of the XuMP pathway, this enzyme was expressed both with and without the peroxisomal targeting sequence. The rational behind this was to ensure that methanol and formaldehyde-assimilating enzymes would both be localised in cytosol of *Y. lipolytica*.

**Fig. 5:**
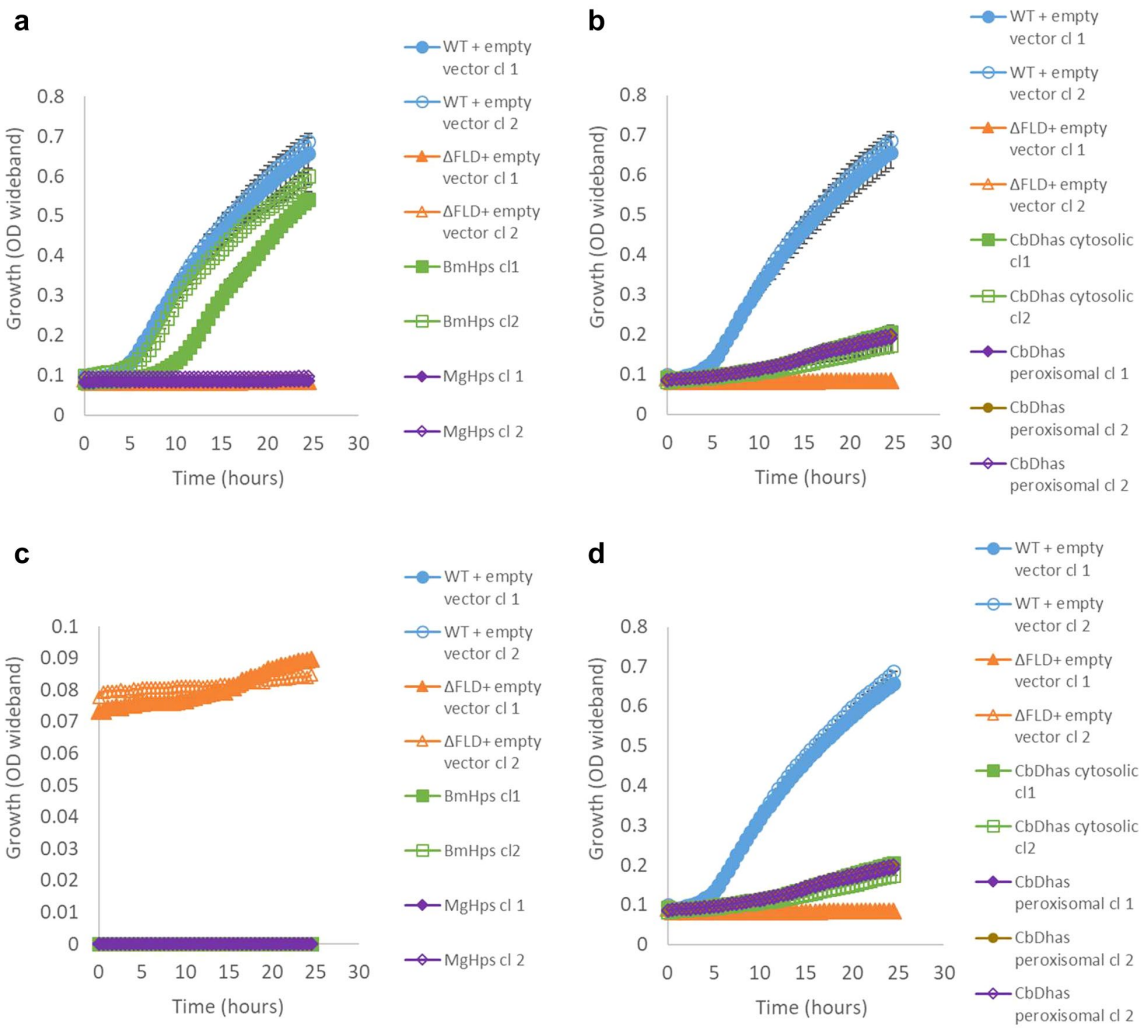
Growth in the presence of 1 mM formaldehyde. **a** HPS strains, **b** DHAS strains, **c** DHAS strains with peroxisomal induction, **d** DHAS strains with peroxisomal induction and without formaldehyde

The formaldehyde tolerance was tested by growing the strains in the presence of 0.1, 0.2, 0.5, 1 and 5 mM formaldehyde, using 0.2 M glycerol as a carbon source. While the Δ*fld1* strain cannot tolerate 1 mM formaldehyde, it was observed that expression of HPS enzyme from *B. methanolicus* was able to rescue the formaldehyde tolerance to wild type level (Fig. 5a). In addition, the expression of DHAS enzyme from *Candida boidinii,* with or without peroxisomal targeting sequence, led to a small increase in the formaldehyde tolerance compared to Δ*fld1* strain (Fig. 5b). Because the removal of peroxisomal targeting sequence is not always enough to change the localisation of a protein, the strains were further tested in the presence of oleic acid that induces peroxisomes. Upon peroxisomal induction by oleic acid, the effect of either type of DHAS enzyme on the formaldehyde tolerance was not anymore observed (Fig. 5c).

### Expression of HPS and PHI enzymes of RuMP pathway in *Y. lipolytica*

As the *B. methanolicus* Hps was shown to rescue the formaldehyde tolerance of the Δ*fld1* strain to the wild type level, the RuMP pathway was chosen to be investigated further. The reaction thermodynamics does not favor methanol oxidation in conditions where *Y. lipolytica* is grown. Thus, a strong pull from HPS reaction would be beneficial for the pathway to work efficiently and a highly active HPS would be required.

**Fig. 6:**
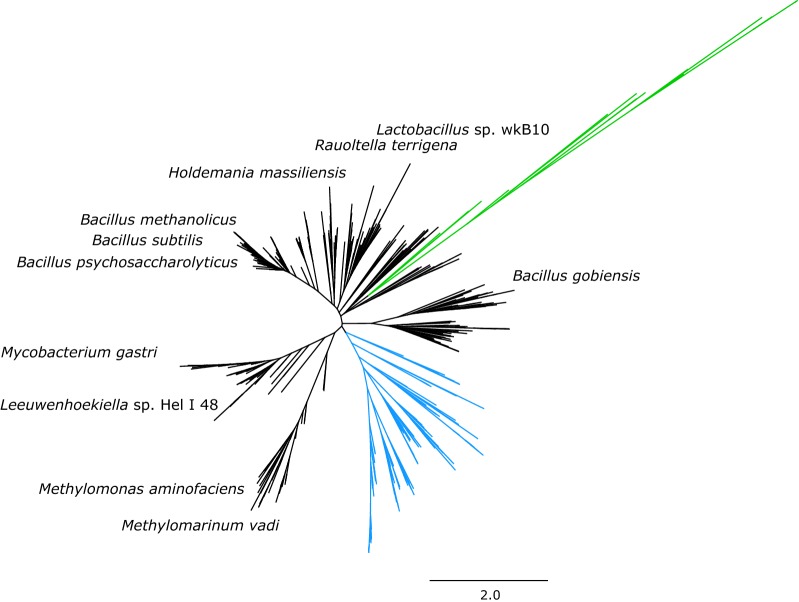
Phylogenetic tree presenting the results of sequence search for HPS enzymes. Proteins which are tested in this paper are marked with the name of the species they are from. Non-HPS members of orotidine 5′-monophosphate decarboxylase (OMPDC) protein family used in the sequence search are marked with green. Archeal proteins are marked with blue. Scale presents the substitutions per site of the sequence alignment

To find optimal HPS enzyme candidates, a sequence search was conducted. Known and predicted members of the orotidine 5′-monophosphate decarboxylase (OMPDC) protein family, to which HPS enzymes belong to, were used as seeds, and candidate enzymes were retrieved (Fig. 6).

**Fig. 7:**
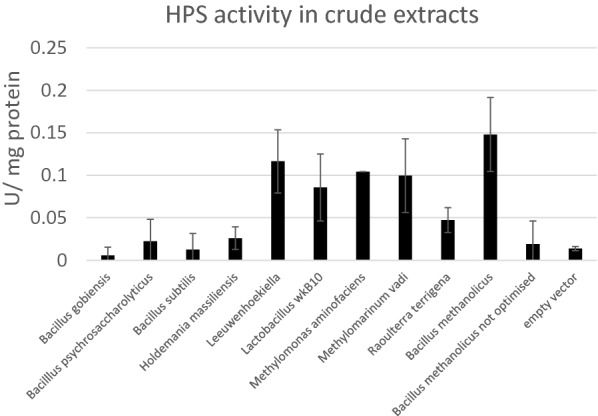
HPS activity in the crude cell extracts of the expression constructs. Duplicate or triplicate measurements for two independent clones of each construct was performed

The predicted sequences were found to be highly similar and all of prokaryotic origin. Nine candidate genes were selected from different branches of the tree and expressed on a centromeric plasmid in the widely used laboratory strain Po1f. In addition, optimised and non-optimised versions of the *B. methanolicus* HPS enzyme were expressed along with empty vector as controls. Seven out of 10 HPS candidates were actively expressed in *Y. lipolytica* and the activity of all top 5 candidates was in the same level (~ 0.1–0.15 U/mg protein) (Fig. 7). None of the candidates exceeded the activity of *B. methanolicus* HPS. Other kinetic properties could, however, differ, but the enzymes were not characterised further.

*Yarrowia lipolytica* optimised HPS and PHI enzymes from *B. methanolicus* were integrated in the FLD locus of the VTT C-00365 strain. The activities of the enzymes in crude cell extracts were 0.15 ± 0.02 U/mg protein and 9.0 ± 2 U/mg protein for HPS and PHI, respectively.

The integrant strain was cultivated in bioreactors in the presence of 0.5 M methanol using either yeast extract or glycerol as a co-substrate. Formaldehyde was produced during the cultivations (Additional file 3: Document S3), but methanol conversion to biomass was not observed under these conditions.

To test if increased copy number would increase the activity of prokaryotic HPS in a eukaryotic host, *B. methanolicus* Hps was expressed in a multicopy vector in *S. cerevisiae*. This expression lead to specific activity of 2.0 ± 0.07 U/mg protein while the centromeric expression in *S. cerevisiae* gave 0.06 ± 0.001 U/mg protein.

## Discussion

Methanol was converted to formaldehyde by *Yarrowia lipolytica* with blocked formaldehyde catabolism pathway and formaldehyde was converted to less toxic intermediates by the action of heterologous HPS or DHAS enzymes of ribulose monophosphate (RuMP) and xylulose monophosphate (XuMP) pathways. However, the use of methanol as carbon source for growth was not observed.

As formaldehyde is a toxic and highly reactive compound, detoxification pathways for it are found in all known organisms [23]. In yeasts, the detoxification pathways for formaldehyde include glutathione dependent formaldehyde dehydrogenase [25]. The formaldehyde dehydrogenase gene was identified from *Y. lipolytica* genome based on the *S. cerevisiae* sequence. The deletion of this gene lead to reduced formaldehyde tolerance. However, the formaldehyde tolerance of this strain was not completely abolished, suggesting that other enzymatic reactions for formaldehyde detoxification may take place in *Y. lipolytica*. These might be, e.g. side activities of aldehyde dehydrogenase as in *Corynebacterium glutamicum* [36].

The formaldehyde dehydrogenase deficient *Y. lipolytica* was able to convert methanol to formaldehyde, most likely by the action of endogenous alcohol dehydrogenases. Formaldehyde production from methanol was observed in two different set ups, in shake flasks without additional carbon source and in bioreactors with glycerol or yeast extract as co-substrate. It has been previously shown that alcohol dehydrogenases of non-methylotrophs are able to use methanol as substrate in vitro [37]. Further, experiments with *C. glutamicum* and *Pseudomonas putida* have shown that the native alcohol dehydrogenases of these organisms can oxidise methanol also in vivo [20, 38].

Three pathways for formaldehyde assimilation were compared in silico by metabolic modeling. As the metabolism of methanol was the ultimate target, the calculations were conducted with the assumption that methanol would be converted to formaldehyde by methanol dehydrogenase reaction. The pathways could not be clearly prioritised based on the modeling and thus it was decided that one prokaryotic and one eukaryotic pathway would be tested. Ribulose monophosphate pathway was chosen as the prokaryotic option due its simplicity compared to serine cycle and because it has been already shown to work in several heterologous hosts [20, 21, 38].

As the methanol conversion to formaldehyde is highly thermodynamically challenging in mesotrophic conditions, a strong pull from formaldehyde-converting enzyme is optimal. Thus, we wanted to ensure that the HPS enzyme chosen for expression would be highly active in eukaryotic host. Completely novel HPS enzymes were searched for and several active ones were found. However, none of them were more active than the enzyme of *B. methanolicus*, which has been used in previous studies. This was probably due to the fact that all the known HPS enzymes are relatively similar and thus the ability of the algorithm to find different types of enzymes is limited.

The expression of *B. methanolicus* HPS enzyme from a centromeric vector was found to rescue the formaldehyde tolerance of the formaldehyde dehydrogenase deficient strain to wild-type level and also the following enzyme, PHI, converting 3-hexulose-6-phosphate to fructose-6P, was expressed in an active form in *Y. lipolytica*. However, although both methanol–formaldehyde and formaldehyde-F6P reactions parts of the pathway were functional when studied separately, no methanol consumption was observed when providing it alone or together with glycerol or yeast extract. The pathway could benefit from higher activity of HPS enzyme, which could be achieved by multicopy expression. *Y. lipolytica* does not possess multicopy plasmids, but insertion to for example rDNA or long terminal repeat loci could be an option to increase expression levels [39]. In addition, boosting of pentose phosphate pathway could enable more efficient recycling of carbon to ribulose-5-phosphate, one of the substrates of HPS enzyme.

Introducing synthetic methylotrophy to other organisms has not been simple either. Introduction of methylotrophic pathways to *E. coli*, *C. glutamicum* and *P. putida* has resulted in relatively modest methanol consumption [20, 21, 38]. During the course of this study, Whitaker et al. (2017) published the first results where function of heterologously expressed methanol assimilation pathway was shown to lead to biomass production from methanol in *E. coli* at 37 °C [40]. The key element in the methanol assimilation was a methanol dehydrogenase with better enzymatic characteristics (low K_m_ for methanol) for methanol oxidation in the temperature where the heterologous host thrives.

Recently, the RuMP pathway from *B. subtilis* was also tested in *S. cerevisiae* [22], although without codon optimisation, but similarly to our results the expression did not result in growth on methanol and was not studied further. However, *S. cerevisiae* was engineered for methanol utilisation by introducing the eukaryotic methylotrophic pathway from *Pichia pastoris* to *S. cerevisiae* [22]. It was shown that expression of alcohol oxidase (AOX), catalase (CAT), dihydroxyacetone synthases (DAS1/2), and dihydroxyacetone kinase (DAK) from *P. pastoris* resulted in consumption of methanol by *S. cerevisiae*. It has been shown that in *P. pastoris*, the whole pathway of methanol assimilation, including the enzymes required for pentose phosphate recycling, exists in the peroxisomes, and the compartmentalisation of formaldehyde assimilation and dissimilation is crucial for growth on methanol in methylotrophic yeasts. Interestingly, the compartmentalisation did not seem to be a problem in *S. cerevisiae* [22].

In our study, the expression of DHAS enzyme from XuMP pathway had only small effect on the formaldehyde tolerance of the formaldehyde dehydrogenase deficient strain. Interestingly, there was no difference in the peroxisomal and cytosolic targeting of the DHAS enzyme. In addition, the induction of peroxisomes by oleic acid abolished the effect of rescuing the formaldehyde tolerance of ∆FLD strain. This suggests that both versions of DHAS enzyme were targeted to peroxisomes despite the removal of the predicted C-terminal signal sequence, which is not uncommon [41]. On glycerol, where the number of peroxisomes is low, some DHAS enzyme would remain in cytosol. On oleic acid, where the number of peroxisomes per cell is considerable larger than on glycerol [42], all the DHAS enzyme would have been in peroxisomes. Thus, the enzyme would have been unable to act on cytosolic formaldehyde.

## Conclusions

Wild type *Yarrowia lipolytica* strain VTT C-00365 was shown to tolerate methanol and thus it emerges as a novel potential host organism that can use crude glycerol as a carbon source. By blocking the formaldehyde dissimilation pathway from this strain, it was shown that methanol is converted to formaldehyde, most likely by the action of native alcohol dehydrogenases.

The formaldehyde dehydrogenase deletion strain was used as tool to study heterologous pathways for formaldehyde assimilation in *Y. lipolytica*. The aim was to introduce into this yeast, a pathway which would assimilate the formaldehyde instead of dissimilating it to carbon dioxide. The RuMP pathway enzymes for formaldehyde assimilation were, indeed, expressed in an active form in this strain, but more work is needed to enable methanol utilisation in crude glycerol streams.

## Methods

### Strains and plasmids

*Yarrowia lipolytica* strains VTT C-00365 and W29 were obtained from the VTT culture collection (http://culturecollection.vtt.fi). Strain Po1f (MATA ura3-302 leu2-270 xpr2-322 axp2-deltaNU49 XPR2::SUC2), derivative of W29, was obtained from ATCC (https://www.atcc.org). In addition, *S. cerevisiae* CEN.PK2-1D (VW-1B; *MAT*α, *leu2*-*3/112 MAL2*-*8cSUC2*) [43] was used as the host strain for HPS expression. *Escherichia coli* strain DH5α [44] was used as the bacterial cloning host.

The expression plasmids were built using the MoClo yeast expression kit [45], Table 2. The parts for *Y.* *lipolytica* were constructed based on the rules for the MoClo kit. All heterologous genes were ordered as synthetic genes or gene fragments from Geneart (Thermo Fisher Scientific Geneart, Germany) and IDT (Integrated DNA Technologies, USA), Table 3. The gene sequences were optimised for *Y. lipolytica* or *S.* *cerevisiae*, depending on the expression host. The optimisation was done either by Geneart or by using the Optimizer-software [46].

**Table 2:** Expression plasmids constructed in this study

Plasmid name	Expression cassette (promoter-gene-terminator)	Selection marker for *Y. lipolytica*
pLif_075	pTpi1-MgHpsPhi-tTef1	Hygromycin
pLif_085	pFba1-CbDhas_tTdh3	Hygromycin
pLif_086	pFba1-CbDhas_syt^a^-tTdh3	Hygromycin
pLif_115	pTpi1-BmHps-tTef1-pTdh3-BmPhi-tPgk1	Hygromycin
pLif_164	pScPgk1-BmHps_Sc-tScAdh1	Leucine
pLif_165	pScPgk1-BmHps_Sc-tScAdh1	Leucine
pLif_166	pFba1-BgHps-tTdh3	Leucine
pLif_167	pFba1-BpHps-tTdh3	Leucine
pLif_168	pFba1-BsHps-tTdh3	Leucine
pLif_169	pFba1-HmHps-tTdh3	Leucine
pLif_170	pFba1-LeeHps-tTdh3	Leucine
pLif_171	pFba1-LbHps-tTdh3	Leucine
pLif_172	pFba1-BgHps-tTdh3	Leucine
pLif_173	pFba1-MvHps-tTdh3	Leucine
pLif_174	pFba1-RtHps-tTdh3	Leucine
pLif_175	pFba1-BmHps_native-tTdh3	Leucine
pLif_176	pFba1-BmHps-tTdh3	Leucine

pLif_165 is a multicopy vector, all other plasmids are centromeric vectors

^a^Cytosolic version of CbDhas was created by omitting the three C-terminal amino acids from the protein

**Table 3:** Synthetic genes used in this study

Gene	Name	NCBI accession number	Origin	Optimised by/with	Optimised for
Dhas	CbDhas	AAC83349	*Candida boidinii*	Geneart	*Y. lipolytica*
Hps	BmHps	WP_003349277	*Bacillus methanolicus*	Geneart	*Y. lipolytica*
Hps	BmHps_native	WP_003349277	*Bacillus methanolicus*	Not optimised	*Y. lipolytica*
Hps	BmHps_Sc	WP_003349277	*Bacillus methanolicus*	Geneart	*S. cerevisiae*
Phi	BmPhi	EIJ79730	*Bacillus methanolicus*	Geneart	*Y. lipolytica*
Phi	BmPhi_Sc	EIJ79730	*Bacillus methanolicus*	Geneart	*S. cerevisiae*
HpsPhi phusion	MgHpsPhi	AB034913	*Mycobacterium gastri*	Geneart	*Y. lipolytica*
Hps	BgHps	WP_053604812	*Bacillus gobiensis*	Optimizer	*Y. lipolytica*
Hps	BpHps	WP_040374929	*Bacillus psychrosaccharolyticus*	Optimizer	*Y. lipolytica*
Hps	BsHps	ASB91925	*Bacillus subtilis*	Optimizer	*Y. lipolytica*
Hps	HmHps	WP_020225866	*Holdemania massiliensis*	Optimizer	*Y. lipolytica*
Hps	LeeHps	WP_028376164	*Leeuwenhoekiella* sp Hel_I_48	Optimizer	*Y. lipolytica*
Hps	LbHps	WP_034981480	*Lactobacillus* wkB10	Optimizer	*Y. lipolytica*
Hps	MaHps	BAA83096	*Methylomonas aminofaciens*	Optimizer	*Y. lipolytica*
Hps	MvHps	WP_031433509	*Methylomarinum vadi*	Optimizer	*Y. lipolytica*
Hps	RtHps	WP_045855207	*Raoulterra terrigena*	Optimizer	*Y. lipolytica*

The integration and deletion cassettes were also built by using the MoClo kit. For the integration of *Bacillus methanolicus* HPS and PHI enzymes to the formaldehyde dehydrogenase (FLD) locus, the RuMP expression cassette from pLif_115 was combined with the nourseotricin selection marker and 1 kb flanking sequences surrounding the FLD open reading frame. For the FLD deletion cassette, the RuMP expression cassette was replaced by a 40 bp spacer sequence from the MoClo-kit.

Native *Y. lipolytica* promoters and terminators were either ordered as synthetic gene blocks or amplified from genomic DNA of the strain VTT C-00365 with primers compatible with the MoClo kit. For centromeric expression, native CEN/ARS68 [47] was amplified by PCR from genomic DNA. In addition, flanking sequences and marker cassettes for hygromycin, nourseotricin and leucine were either obtained as synthetic gene fragments or constructed with the MoClo kit. The sequences of all gene elements are presented in Additional file 4: Table S1. All components of the *S. cerevisiae* plasmids, other than the HPS and the PHI open reading frames, originated from the MoClo kit.

Transformations of *Y. lipolytica and S. cerevisiae* were done using Frozen-EZ yeast transformation kit II (Zymo Research, USA).

### Culture conditions

Small scale cultivations were performed with Bioscreen microtiter plate cultivator (Growth Curves Ltd, Finland). Synthetic minimal medium (yeast nitrogen base without amino acids), containing 5 g/L (0.04 M) (NH_4_)_2_·SO_4_ and with addition of selected carbon source was used in all small scale cultivations. The following Bioscreen settings were used: temperature + 30 °C, continuous shaking, maximum speed, measurement interval 30 min, filter 420–580 nm.

Bioreactor cultivations were performed in 15-unit bioreactor (Medicel, Finland) in the defined minimal medium described by Verdyun et al. [48], with 5 g/L (0.05 M) glycerol or 10 g/L yeast extract and 16 g/L (0.5 M) methanol as carbon source. BDH silicon antifoam was used to prevent foaming of the cultures. The culture conditions were as follows: 200 mL volume, pH 5.0, + 30 °C and with 0.5 vvm air.

### Enzyme activity assays

Enzyme activities were measured as units (U) per mg of total soluble protein. One U was defined as the activity which converts one µmol substrate per min. Enzyme activities were measured from cell extracts prepared by disrupting the yeast cells with glass beads in 100 mM sodium phosphate buffer, pH 7.6, supplemented with Complete protease inhibitor cocktail (Roche Applied Science, USA). The protein concentration of the extracts was determined with Bio-Rad Protein Assay (Bio-Rad Laboratories, USA), using immunoglobulin G as the standard.

Methanol dehydrogenase and 6-phospho-3-hexulose isomerase were assayed using Konelab Arena 20XT automated analyser (Thermo Scientific, Finland) at 30 °C and 340 nm. Methanol dehydrogenase (EC 1.1.1.244) reaction mixture contained 100 mM Tris–HCl, pH 9.5, 5 mM MgSO_4_ and 0.5 mM NAD. The reaction was started by adding methanol or ethanol to a final concentration of 500 mM. 6-phospho-3-hexulose isomerase (EC 5.3.1.27) reaction mixture contained 50 mM sodium phosphate buffer, pH 7.6, 5 mM MgCl_2_, 5 mM ribose-5-phosphate, 2.5 mM NADP, 5 U/mL phosphoribose isomerase (PRI) (Sigma-Aldrich, USA), 5 U/mL glucose-6-phosphate dehydrogenase (G6PDH) (Sigma-Aldrich, USA), 5 U/mL phoshoglucose isomerase (PGI) (Sigma-Aldrich, USA) and ~ 0.01 U of recombinant 3-hexulose-6-phosphate synthase (HPS) purified from *E. coli* overproducing *Methylomonas aminofaciens* HPS. The reaction was started by adding formaldehyde to a final concentration of 5 mM.

3-Hexulose-6-phosphate synthase (HPS) activity was determined in a discontinuous assay by measuring the disappearance of formaldehyde. The reaction mixture contained 100 mM sodium phosphate buffer, pH 7.6, 5 mM MgCl_2_, 5 mM formaldehyde, 5 mM ribose-5-phosphate and 17 U/mL phosphoribose isomerase (Sigma-Aldrich, USA). The mixture was preincubated at 37 °C for 15 min, at 30 °C for 5 min and the reaction was started by addition of crude cell extract. Samples were taken every 60 s and mixed with 0.1 M HCl solution to stop the enzymatic activity. The formaldehyde concentration in the samples was determined with NASH assay [49]. Briefly, the sample dilutions were mixed with freshly made NASH assay mixture containing 2 M ammonium acetate, 50 mM acetic acid and 20 mM acetylacetone. The mixture was incubated 1–2 h at room temperature and measured at 412 nm with Varioskan mictrotiter plate reader (Thermo Fischer Scientific, USA). 5–500 µM formaldehyde solutions were used to prepare the standard curve.

### Formaldehyde production experiments

For formaldehyde production assay, yeast cells were grown in 2 mL of yeast peptone (YP)-medium, containing 0.2 M glycerol for 24 h (+ 28 °C, 250 rpm, 80% humidity), washed with synthetic minimal medium and suspended in 2 ml volume of the same medium at OD_600_ of 10. The experiment was started by the addition of methanol to a final concentration of 0.5 M. During the experiment, the cells were kept in the incubator (+ 28 °C, 250 rpm, 80% humidity). Every 15 min 200 µL samples were withdrawn and centrifuged for 1 min at 15,000×*g*. Supernatants were analysed for formaldehyde concentration with NASH reagent as described in HPS assay.

### Sequence search for new HPS candidates

To identify potential genes encoding 3-hexulose-6-phosphate synthases (HPS), a protein BLAST search against the NCBI nr database (E-value < 1e−5; http://blast.ncbi.nlm.nih.gov) was carried out using seventeen query sequences for known and predicted members of the orotidine 5′-monophosphate decarboxylase (OMPDC) protein family. The query sequences used were; *Mycobacterium gastri* HPS (Q9LBW4), *Salmonella typhimurium* HPS (Q8ZMP9), *Bacillus methanolicus* HPS (I3DZR0), *Methylomonas aminofaciens* HPS (Q48907), *E. coli* 3-keto-l-gulonate-6-phosphate decarboxylase (P39304; PDB1KV8), Human Uridine 5′-monophosphate synthase (P11172; PDB2EAW), *Vibrio cholerae serotype O1* putative HPS (Q9KMS8; PDB3IEB), *Metallosphaera sedula* OMPDC (A4YI54; PDB3VE7), *Leishmania infantum* OMPDC (A4HWV2; PDB3QW3), *Toxoplasma gondii* OMPDC (B6KBH9; PDB4MJZ), *E. coli* OMPDC (P08244; PDB1EIX), *Plasmodium falciparum* OMPDC (Q8IJH3; PDB2F84), *Streptomyces avermitilis* OMPDC (Q827Q5; PDB3V75), *Saccharomyces cerevisiae* OMPDC (P03962; PDB1DQW), *Plasmodium yoelii yoelii* putative OMPDC (Q7RPE4; PDB2AQW), *Plasmodium berghei* putative OMPDC (Q4Z4C3; PDB2FDS), and *Methanothermobacter thermautotrophicus* OMPDC (O26232; PDB1DV7) (the identifiers refer to UniProt, the Universe protein resource, available at http://www.uniprot.org/ and, if available to PDB, the Protein Data Bank, available at http://www.rcsb.org/pdb/home/home.do). To remove redundancy among the retrieved sequences they were clustered using BLASTclust (http://blast.ncbi.nlm.nih.gov) to groups in which members are at most 90% identical to each other. After clustering and addition of the query sequences and manually selected sequences, a set of 576 sequences remained which were aligned to the orotidine 5′-monophosphate decarboxylase Pfam motif (PF00215) using HMMER (http://hmmer.janelia.org/). Sequences that were not full-length aligned to the Pfam (PF00215) motif were removed. Phylogenetic tree reconstruction was performed using the Tree Builder of the Geneious program version 5.5.3 with default parameters using the Neighbour-Joining algorithm.

### Modeling

The *Y. lipolytica* metabolic model iNL895 [50] was used in all calculations. The model is based on the *S.* *cerevisiae* models iMM904 [51], iN800 [52], and consensus model 4.36 [53]. The current consensus model of *S. cerevisiae* at the time when this work was conducted was yeast 7.6. The *Y. lipolytica* model iNL895 was modified based on the newest *S. cerevisiae* model and other literature (Additional file 2: Document S1).

## Supplementary information

**Additional file 1: Document S1.** Reconciliation of thermodynamic data for formaldehyde.

**Additional file 2: Document S2.** Modification to genome scale model iNL895.

**Additional file 3: Document S3.** Extracellular metabolites analysed from bioreactor cultivations.

**Additional file 4: Table S1.** Sequences of synthetic gene fragments and those of PCR oligos for amplification of promoters, terminators and ARS.

## Data Availability

All main data generated or analysed during this study are included in this published article and its additional files. The raw data used are available from the corresponding author on reasonable request.

## Abbreviations

SCP: single cell protein
EPA: omega-3-eicosapentaenoic acid
XuMP: xylulose monophosphate pathway
RuMP: ribulose monophosphate pathway
TAG: triacylglycerol
OMPDC: orotidine 5′-monophosphate decarboxylase
MoClo: modular cloning
YP: yeast peptone

## References

[1] Wolf K (1996). Nonconventional yeasts in biotechnology: a handbook.

[2] Coelho MAZ, Amaral PFF, Belo I, Méndez-Vilas A (2010). Yarrowia lipolytica: an industrial workhorse. Current research, technology and education topics in applied microbiology and microbial biotechnology advances.

[3] Madzak C (2015). Yarrowia lipolytica: recent achievements in heterologous protein expression and pathway engineering. Appl Microbiol Biotechnol.

[4] Xie D, Jackson EN, Zhu Q (2015). Sustainable source of omega-3 eicosapentaenoic acid from metabolically engineered *Yarrowia lipolytica*: from fundamental research to commercial production. Appl Microbiol Biotechnol.

[5] Juszczyk P, Wojtatowicz M, Robak M, Lazar Z, Rywi A, Tomaszewska L, Rymowicz W (2013). Glycerol as a promising substrate for *Yarrowia lipolytica* biotechnological applications. Biomass Bioenergy.

[6] Canonico L, Ashoor S, Taccari M, Comitini F, Antonucci M, Truzzi C (2016). Conversion of raw glycerol to microbial lipids by new Metschnikowia and *Yarrowia lipolytica* strains. Ann Microbiol.

[7] Hoydonckx HE, Dirk E, Chavan SA, Jacobs PA (2004). Esterification and transesterification of renewable chemicals. Top Catal.

[8] Meher LC, Sagar DV, Naik SN (2006). Technical aspects of biodiesel production by transesterification—a review. Renew Sustain Energy Rev.

[9] Johnson DT, Taconi KA (2009). The glycerin glut: options for the value-added conversion of crude glycerol resulting from biodiesel production. Environ Prog.

[10] Bevilacqua A, Aragão-leoneti V, Valle S, Borges W, Oliveira D (2012). Glycerol as a by-product of biodiesel production in Brazil: alternatives for the use of unrefined glycerol. Renew Energy.

[11] Uçkun E, Trzcinski A, Webb C (2013). Microbial oil produced from biodiesel by-products could enhance overall production. Biores Technol.

[12] Xiao Y, Xiao G, Varma A (2013). A universal procedure for crude glycerol purification from different feedstocks in biodiesel production: experimental and simulation study. Ind Eng Chem Res.

[13] Yang F, Hanna MA, Sun R (2012). Value-added uses for crude glycerol—a byproduct of biodiesel production. Biotechnol Biofuels.

[14] Garlapati VK, Shankar U, Budhiraja A (2016). Bioconversion technologies of crude glycerol to value added industrial products. Biotechnol Rep.

[15] Pyle DJ, Garcia RA, Wen Z (2008). Producing docosahexaenoic acid (DHA)-rich algae from biodiesel-derived crude glycerol: effects of impurities on DHA production and algal biomass composition. J Agric Food Chem.

[16] Salakkam A, Webb C (2015). The inhibition effect of methanol, as a component of crude glycerol, on the growth rate of *Cupriavidus necator* and other micro-organisms. Biochem Eng J.

[17] Thompson JC, He BB (2006). Characterization of crude glycerol from biodiesel production from multiple feedstocks. Appl Eng Agric.

[18] Iyyappan J, Bharathiraja B, Baskar G, Jayamuthunagai J, Barathkumar S, Anna R (2018). Malic acid production by chemically induced *Aspergillus niger* MTCC 281 mutant from crude glycerol. Biores Technol.

[19] Whitaker WB, Sandoval NR, Bennett RK, Fast AG, Papoutsakis ET (2015). Synthetic methylotrophy: engineering the production of biofuels and chemicals based on the biology of aerobic methanol utilization. Curr Opin Biotechnol.

[20] Witthoff S, Schmitz K, Niedenführ S, Nöh K, Noack S, Bott M, Marienhagen J (2015). Metabolic engineering of *Corynebacterium glutamicum* for methanol metabolism. Appl Environ Microbiol.

[21] Müller JEN, Meyer F, Litsanov B, Kiefer P, Potthoff E, Heux S (2015). Engineering *Escherichia coli* for methanol conversion. Metab Eng.

[22] Dai Z, Gu H, Zhang S, Xin F, Zhang W, Dong W (2017). Metabolic construction strategies for direct methanol utilization in *Saccharomyces cerevisiae*. Biores Technol.

[23] Chen NH, Djoko KY, Veyrier FJ, McEwan AG (2016). Formaldehyde stress responses in bacterial pathogens. Front Microbiol.

[24] Mason RP, Sanders JK, Crawford A, Hunter BK (1986). Formaldehyde Metabolism by *Escherichia coli*. Detection by in Vivo 13C NMR spectroscopy of s-(hydroxymethyl)glutathione as a transient intracellular intermediate. Biochemistry.

[25] Lee B, Yurimoto H, Sakai Y, Kato N (2002). Physiological role of the glutathione-dependent formaldehyde dehydrogenase in the methylotrophic yeast *Candida boidinii*. Microbiology.

[26] Overkamp KM, Kötter P, van der Hoek R, Schoondermark-Stolk S, Luttik MH, van Dijken JP, Pronk JT (2002). Functional analysis of structural genes for NAD+ -dependent formate dehydrogenase in *Saccharomyces cerevisiae*. Yeast.

[27] Weimer EP, Rao E, Brendel M (1993). Molecular structure and genetic regulation of SFA, a gene responsible for resistance to formaldehyde in *Saccharomyces cerevisiae*, and characterization of its protein product. Mol Gen Genetics MGG..

[28] Chistoserdova LV, Lidstrom ME (1994). Genetics of the serine cycle in [i]Methylobacterium extorquens[/i] AM1: identification of sgaA and mtdA and sequences of sgaA, hprA, and mtdA. J Bacteriol.

[29] Kato N, Yurimoto H, Thauer RK (2006). The physiological role of the ribulose monophosphate pathway in bacteria and archaea. Biosci Biotechnol Biochem.

[30] Anthony C (2011). How half a century of research was required to understand bacterial growth on C1 and C2 compounds; the story of the serine cycle and the ethylmalonyl-CoA pathway. Sci Prog.

[31] Britain G, Kemp MB (1974). Hexose phosphate synthase from *Methylococcus capsulatus* makes d-arabino-3-hexulose phosphate. Biochem J..

[32] Ferenci T, Strom T, Quayle JR (1974). Purification and properties of 3-hexulose phosphate synthase and phospho-3-hexuloisomerase from Methylococcus capsulatus. Biochem J.

[33] van Dijken JP, Harder W, Beardsmore AJ, Quayle JR (1978). Dihydroxyacetone: an intermediate in the assimilation of methanol by yeasts?. FEMS Microbiol Lett.

[34] Rußmayer H, Buchetics M, Gruber C, Valli M, Grillitsch K, Modarres G (2015). Systems-level organization of yeast methylotrophic lifestyle. BMC Biol.

[35] Rivlin M, Eliav U, Navon G (2015). NMR studies of the equilibria and reaction rates in aqueous solutions of formaldehyde. J Phys Chem B..

[36] Leßmeier L, Pfeifenschneider J, Carnicer M, Heux S, Portais JC, Wendisch VF (2015). Production of carbon-13-labeled cadaverine by engineered *Corynebacterium glutamicum* using carbon-13-labeled methanol as co-substrate. Appl Microbiol Biotechnol.

[37] Mani JC, Pietruszko R, Theorell H (1970). Methanol activity of alcohol dehydrogenases from human liver, horse liver, and yeast. Arch Biochem Biophys.

[38] Koopman FW, de Winde JH, Ruijssenaars HJ (2009). C1 compounds as auxiliary substrate for engineered *Pseudomonas putida* S12. Appl Microbiol Biotechnol.

[39] Juretzek T, Le Dall M, Mauersberger S, Gaillardin C, Barth G, Nicaud J (2001). Vectors for gene expression and amplification in the yeast *Yarrowia lipolytica*. Yeast (Chichester, England)..

[40] Price JV, Chen L, Whitaker WB, Papoutsakis E, Chen W (2016). Scaffoldless engineered enzyme assembly for enhanced methanol utilization. Proc Natl Acad Sci.

[41] Brocard C, Hartig A (2006). Peroxisome targeting signal 1: is it really a simple tripeptide?. Biochem Biophys Acta.

[42] Eitzen A, Szilard KR, Rachubinski RA (1997). Enlarged peroxisomes are present in oleic acid–grown *Yarrowia lipolytica* overexpressing the PEX16 gene encoding an intraperoxisomal peripheral membrane peroxin. J Cell Biol.

[43] Bolesm E, Göhlmann HWH, Zimmermann FK (1996). Cloning of a second gene encoding 6-phosphofructo-2-kinase in yeast, and characterization of mutant strains without fructose-2,6-bisphosphate. Mol Microbiol.

[44] Woodcock DMCPDJ (1989). Quantitative evaluation of *Escherichia coli* host strains for tolerance tocytosine methylation in plasmid and phage recombinants. Nucleic Acids Res.

[45] Lee ME, DeLoache WC, Cervantes B, Dueber JE (2015). A highly characterized yeast toolkit for modular, multipart assembly. ACS Syn Biol.

[46] Puigbò P, Guzmán E, Romeu A, Garcia-Vallvé S (2007). OPTIMIZER: a web server for optimizing the codon usage of DNA sequences. Nucleic Acids Res.

[47] Fournier P, Abbas A, Chasles M, Kudla B, Ogrydziak DM, Yaver D (1993). Colocalization of centromeric and replicative functions on autonomously replicating sequences isolated from the yeast *Yarrowia lipolytica*. Proc Natl Acad Sci USA.

[48] Verduyn C, Postma E, Scheffers WA, van Dijken JP (1992). Effect of benzoic acid on metabolic fluxes in yeasts: a continuous-culture study on the regulation of respiration and alcoholic fermentation. Yeast.

[49] Nash T (1953). The colorimetric estimation of formaldehyde by means of the Hantzsch reaction. Biochem J.

[50] Loira N, Dulermo T, Nicaud J, Sherman D (2012). A genome-scale metabolic model of the lipid-accumulating yeast *Yarrowia lipolytica*. BMC Syst Biol.

[51] Mo ML, Palsson B, Herrgaard MJ (2009). Connecting extracellular metabolomic measurements to intracellular flux states in yeast. BMC Syst Biol.

[52] Nookaew I, Jewett MC, Meechai A, Thammarongtham C, Laoteng K, Cheevadhanarak S (2008). The genome-scale metabolic model iIN800 of *Saccharomyces cerevisiae* and its validation: a scaffold to query lipid metabolism. BMC Syst Biol.

[53] Herrgård MJ, Swainston N, Dobson P, Dunn WB, Arga KY, Arvas M (2008). A consensus yeast metabolic network reconstruction obtained from a community approach to systems biology. Nat Biotechnol.

