# Supplementary material for: Evaluation of synthetic formaldehyde and methanol assimilation pathways in Yarrowia lipolytica
Source: Fungal Biol Biotechnol. 2019 Dec 17;6:27. doi: 10.1186/s40694-019-0090-9 (PMC6918578; doi:10.1186/s40694-019-0090-9)
Supplement: Supplementary file 1 — Additional file 1: Document S1. Reconciliation of thermodynamic data for formaldehyde. [file 40694_2019_90_MOESM1_ESM.docx]

# Calculating the maximum formaldehyde concentration

The maximum formaldehyde concentration can be estimated based on equilibrium thermodynamics.

The enzyme-catalyzed formation of formaldehyde from methanol is a 2-electron oxidation reaction with NAD as the cofactor .

The apparent concentration of hydrolyzed formaldehyde can be estimated from equation once the concentration of methanol and the NAD/NADH-ratio are known.

$c_{\text{FAld}}\approx\frac{K_{\text{eq}}c_{\text{MeOH}}c_{\text{NAD}}}{c_{\text{NADH}}}$,

where the equilibrium constant Keq is calculated from

$$-RT\ln K_{\text{eq}}=\Delta_{\text{r}}G_{\text{NET}}^{T}=\Delta_{\text{r}}G_{\text{MeOH}}^{T}+\Delta_{\text{r}}G_{\text{NAD}}^{T}=-nF\varepsilon_{\text{MeOH}}-nF\varepsilon_{\text{NAD}}=-nF\varepsilon_{\text{NET}}$$

The enzyme-catalyzed reaction can be split into two half-reactions; and .

$$\text{NAD}\left(\text{aq}\right)+2\text{H}^{+}+2\text{e}^{-}\xrightleftharpoons{}\text{NADH}\left(\text{aq}\right)$$

$$\text{MeOH}\left(\text{aq}\right)\xrightleftharpoons{}\text{FAld}\left(\text{aq}\right)+2\text{e}^{-}+2\text{H}^{+}\left(\text{aq}\right)$$

The temperature dependence of the apparent reduction potential of the NADH/NAD redox couple, -0.00131 V K^-1^, was acquired from [^[[1]](#endnote-2)^]. The standard reduction potential of NAD at 25°C and pH 7 was reported as -0.320 ± 0.004 V vs hydrogen electrode [1, ^[[2]](#endnote-3)^].

The calculation of the Gibbs energy of reaction at temperatures other than the standard temperature 25°C requires the enthalpy, the entropy, the temperature-dependent heat capacity, and the stoichiometry of reaction. First, the entropy of formation is calculated with equation , then the enthalpies and entropies of formation are updated to the desired temperature with equations and , and finally, the Gibbs energy of reaction is calculated with equations and .

$$\Delta_{\text{f}}S_{k}^{298\text{K}}=\frac{\Delta_{\text{f}}H_{k}^{298\text{K}}-\Delta_{\text{f}}G_{k}^{298\text{K}}}{T^{298\text{K}}}$$

$$\Delta_{\text{f}}H_{k}^{T}=\Delta_{\text{f}}H_{k}^{298\text{K}}+\int_{298\text{K}}^{T} C_{\text{p},k}\left(T\right)dT$$

$$\Delta_{\text{f}}S_{k}^{T}=\Delta_{\text{f}}S_{k}^{298\text{K}}+\int_{298\text{K}}^{T} \frac{C_{\text{p},k}\left(T\right)}{T}dT$$

$$\Delta_{\text{f}}G_{k}^{T}=\Delta_{\text{f}}H_{k}^{T}-T\Delta_{\text{f}}S_{k}^{T}$$

$$\Delta_{\text{r}}G^{T}=\sum_{k} \nu_{k}\Delta_{\text{f}}G_{k}^{T}$$

The pertinent thermodynamic property data for the methanol and formaldehyde half reaction is shown in Table 1. Heat capacity data is derived from [^[[3]](#endnote-4)^]. The Gibbs energy and enthalpy of formation for methanol is listed in [^[[4]](#endnote-5)^]. The Gibbs energy and enthalpy of formation values for hydrolyzed formaldehyde are based on this work. The heat capacity parameters A, B, C, and D correspond to expression .

Table 1. Thermodynamic property values for the methanol and hydrolyzed formaldehyde half reaction. The Gibbs energy of formation and the enthalpy of formation values for hydrolyzed formaldehyde are based on this work.

|  | **Methanol** | **Hydrolyzed formaldehyde** | **Hydrogen ion** |
| --- | --- | --- | --- |
| $\Delta_{\text{f}}G_{k}^{298\text{K}}$[kJ mol^-1^] | -175.234 | -130.54 | 0 |
| $\Delta_{\text{f}}H_{k}^{298\text{K}}$[kJ mol^-1^] | -245.977 | -171.16 | 0 |
| $\Delta_{\text{f}}S_{k}^{298\text{K}}$[kJ mol^-1^ K^-1^] | -0.237273 | -0.13624 | 0 |
| $c_{k}^{\text{ref}}$ [M] | 1 | 1 | 1 (pH = 0) |
| $\nu_{k}$ | -1 | 1 | 2 |
| A [J mol^-1^ K^-1^] | -1016.630412 | 10696.67214 | 0 |
| B | 4.498578224 | -43.96927944 | 0 |
| D | 23659976.08 | -187141701 | 0 |
| C | -0.00487013 | 0.051567382 | 0 |

$$C_{\text{p},k}\left(T\right)=A+BT+CT^{2}+\frac{D}{T^{2}}$$

The Gibbs energy of formation for hydrogen ions at pH other than the reference pH, pHref = 0, is calculated by equation .

$$\Delta_{\text{f}}G_{\text{H}}\left(pH\right)=RT\ln\left(10^{-pH}\right)$$

# Reconciliation of thermodynamic data for formaldehyde

The Gibbs energy of formation and the enthalpy of formation for aqueous and gaseous formaldehyde reported in literature show great variability. For gaseous formaldehyde, published values for the Gibbs energy of formation range from -102.53 kJ mol^-1^ to -113.49 kJ mol^-1^, while the values for the enthalpy of formation range from -103.35 kJ mol^-1^ to -118 kJ mol^-1^. For aqueous formaldehyde, Gibbs energies of formation listed in literature range from -109.33 kJ mol^-1^ to -133.89 kJ mol^-1^, while the values for the enthalpy of formation range from -141.8 kJ mol^-1^ to -179.91 kJ mol^-1^. The reported values strongly cluster at the boundaries of the ranges, thus indicating a systematic difference rather than random uncertainty.

The accurate determination of thermodynamic parameters for aqueous formaldehyde is hampered by the spontaneous hydrolysis of formaldehyde to methylene glycol [^[[5]](#endnote-6)^, ^[[6]](#endnote-7)^].

$$\text{FAld}\left(\text{aq}\right)+\text{H}_{2}\text{O}\left(\text{liq}\right)\rightleftarrows\text{MG}\left(\text{aq}\right)$$

At equilibrium, formaldehyde in aqueous solutions is almost fully hydrolyzed to methylene glycol [^[[7]](#endnote-8)^, ^[[8]](#endnote-9)^, ^[[9]](#endnote-10)^]. The equilibrium ratio can be shifted in favour of formaldehyde by increasing the pH and by increasing the temperature [6, 7, 8, 9].

In the NBS tables of chemical thermodynamic properties from 1982 [4], the term hydrolyzed formaldehyde denotes an equilibrium mixture of formaldehyde and methylene glycol treated as if it were formaldehyde. In modern terms, it is formally expressed with a Legendre transform .

$G'=G-n_{\text{w}}G_{\text{w}}$,

where $G'$ is the transformed Gibbs energy, G the Gibbs energy, nw the number of occurrences of ‘water’ in the molecule, and Gw the Gibbs energy of ‘water’. For formaldehyde and methylene glycol, nw is 0 and 1, respectively. The transformation implies that formaldehyde and methylene glycol are in equilibrium. Thus, they can be treated as one entity, i.e. as hydrolyzed formaldehyde.

Since almost all formaldehyde is hydrolyzed to methylene glycol at equilibrium, methylene glycol is almost the same as hydrolyzed formaldehyde, except for the contribution of ‘water’ in the Legendre transform.

Table 2. Reconciled thermodynamic property data for formaldehyde and methylene glycol in aqueous and gas phases at 298.15 K. The uncertainties are generally below 3 kJ mol^-1^.

| **Molecule** | **Phase** | **Thermodynamic property** | **Approximate value**  [kJ mol^-1^] |
| --- | --- | --- | --- |
| Formaldehyde | Aqueous | Gibbs energy of formation | -109 |
| Formaldehyde | Aqueous | Enthalpy of formation | -142 |
| Formaldehyde | Gas | Gibbs energy of formation | -103 |
| Formaldehyde | Gas | Enthalpy of formation | -109 |
| Methylene glycol | Aqueous | Gibbs energy of formation | -130 |
| Methylene glycol | Aqueous | Enthalpy of formation | -171 |
| Methylene glycol | Gas | Gibbs energy of formation | -110 |
| Methylene glycol | Gas | Enthalpy of formation | -116 |

The enthalpy of formation for anhydrous formaldehyde gas at 298.15 K was recently measured with high accuracy and validated with reaction equilibrium data to a value of -108.99 kJ mol^-1^ ± 1.76 kJ mol^-1^ [^[[10]](#endnote-11)^]. The NBS tables from 1982 listed the enthalpies of formation for aqueous formaldehyde -141.8 kJ mol^-1^ and aqueous hydrolyzed formaldehyde -170.7 kJ mol^-1^ [4]. The latter value has since been refined to -171.16 ± 0.59 kJ mol^-1^ [^[[11]](#endnote-12)^] based on hydration enthalpy measurements. The difference, -62.17 kJ mol^-1^, is in very close agreement with the pioneering enthalpy measurement for the dissolution of anhydrous formaldehyde gas into water, -61.92 kJ mol^-1^ [^[[12]](#endnote-13)^], for which the uncertainty is estimated to be ± 0.8 kJ mol^-1^ [6].

Based on chemical reaction data, Thauer *et al.* obtained a value of -130.54 kJ mol^-1^ for the Gibbs energy of formation for aqueous methylene glycol [^[[13]](#endnote-14)^]. The Gibbs energy of formation for gaseous methylene glycol has been calculated from enthalpy, entropy, and heat capacity data to be -109.9 kJ mol^-1^ at 298.15 K [^[[14]](#endnote-15)^, ^[[15]](#endnote-16)^, ^[[16]](#endnote-17)^]. The recommended value for the Henry law coefficient of methylene glycol, 3230 M atm^-1^ [^[[17]](#endnote-18)^], corresponds to a Gibbs energy difference of 20.03 kJ mol^-1^. The Gibbs energy of formation for aqueous methylene glycol estimated from the Gibbs energy of formation for gaseous methylene glycol and the Henry law coefficient becomes -129.95 kJ mol^-1^. This value is in reasonable agreement with the value (-130.54 kJ mol^-1^) tabularized by Thauer *et al.*.

The NBS tables of chemical thermodynamic properties from 1982 lists the enthalpy of hydrolysis in water as -28.9 kJ mol^-1^ [4]. More recently, Winkelman et al. measured the hydrolysis kinetics of aqueous formaldehyde to methylene glycol and obtained -31.4 kJ mol^-1^ for the standard enthalpy of reaction [^[[18]](#endnote-19)^]. This value agrees with the value (-28.9 kJ mol^-1^) calculated from the NBS tables of chemical thermodynamic properties from 1982. Based on the work of Winkelman et al., Golden and Valentini concluded that methylene glycol and formaldehyde coexist in aqueous solutions as a dynamic equilibrium with a ratio of 2499:1 at standard temperature and pressure and neutral pH [9]. The ratio corresponds to a transformed Gibbs energy difference of 19.4 kJ mol^-1^.

These property values are summarized in Table 3.

Table 3. Measured thermodynamic data for select reactions. The molecule assignments for the reactants are based on this work.

| Reference | Reaction | Property | Value  kJ mol^-1^ | Comment |
| --- | --- | --- | --- | --- |
| 12 | $\text{FAld}\left(\text{g}\right)\to\text{Hyd}\left(\text{aq}\right)$ | Enthalpy | -61.92 |  |
| 10 | $\text{FAld}\left(\text{aq}\right)\to\text{Hyd}\left(\text{aq}\right)$ | Enthalpy | -28.9 |  |
| 18 | $\text{FAld}\left(\text{aq}\right)\to\text{MG}\left(\text{aq}\right)$ | Enthalpy | -31.4 | Based on kinetics |
| 9 | $\text{FAld}\left(\text{aq}\right)\rightleftarrows\text{MG}\left(\text{aq}\right)$ | Gibbs energy | -19.39 | Ratio 2499:1 |
| 17 | $\text{Hyd}\left(\text{g}\right)\to\text{Hyd}\left(\text{aq}\right)$ | Gibbs energy | -20.03 | Henry law coefficient 3230 M atm^-1^ |

## Thermodynamic data for formaldehyde and methanol

The NBS tables list the Gibbs energy of formation and the enthalpy of formation for gaseous formaldehyde as -102.53 kJ mol^-1^ and -108.57 kJ mol^-1^, respectively [4]. The enthalpy of formation for gaseous formaldehyde was recently measured and validated by reactions to -108.99 ± 1.76 kJ mol^-1^ [^[[19]](#endnote-20)^].

A similar compilation of standard thermodynamic properties that accounted for measured reaction data listed the Gibbs energies of formation for aqueous and gaseous hydrolyzed formaldehyde as -130.54 kJ mol^-1^ and -112.97 kJ mol^-1^, respectively [13].

The enthalpy of formation for moist formaldehyde vapor has caused confusion in the past as it has been listed as formaldehyde, but with value approximately 7 kJ mol^-1^ more negative than the value for anhydrous formaldehyde vapor. The enthalpy of formation for moist formaldehyde vapor -115.897 kJ mol^-1^ was reported as early as 1944 [6].

The Gibbs energy of formation and the enthalpy of formation for aqueous methanol are provided by reference [4] as -175.23 kJ mol^-1^ and -245.98 kJ mol^-1^, respectively.

Table 4 compiles an extended list of pertinent thermodynamic values for formaldehyde and methanol.

Table 4. Transformed Gibbs energies of formation and transformed enthalpies of formation at standard state (298.15 K) collected from literature. The molecule assignments are based on this work. HF = Hydrolyzed formaldehyde

| Reference | Molecule | Phase | $\Delta_{\text{f}}G'_{298\text{K}}^{○}$  kJ mol^-1^ | $\Delta_{\text{f}}H'_{298\text{K}}^{○}$  kJ mol^-1^ | Comment |
| --- | --- | --- | --- | --- | --- |
| ^[[20]](#endnote-21)^ | Formaldehyde | aq | -109.328 | -141.796 |  |
| 4 | Formaldehyde | aq |  | -141.8 |  |
| ^[[21]](#endnote-22)^ | Formaldehyde | aq | -109.327 | -145.03 |  |
| 4, ^[[22]](#endnote-23)^ | Formaldehyde | g | -102.53 | -108.57 |  |
| 14 | Formaldehyde | g |  | -108.6 | ±0.46 |
| ^[[23]](#endnote-24)^ | Formaldehyde | g | -102.649 | -108.7 |  |
| 10 | Formaldehyde | g |  | -108.99 | ±1.76 |
| ^[[24]](#endnote-25)^ | Formaldehyde | g |  | -109.188 | ±0.099 |
| ^[[25]](#endnote-26)^ | Formaldehyde | g |  | -109.23 |  |
| 4 | HF | aq |  | -170.7 | hydrolyzed |
| 11 | HF* | aq |  | -171.16 | ±0.59 |
| 13 | HF* | aq | -130.54 |  |  |
| 6, 10 | HF* | g |  | -115.897 |  |
| 14 | HF* | g | -109.921 | -115.897 |  |
| 15 | HF* | g | -110 | -115.9 |  |
| 16 | HF* | g | -109.939 | -115.9 |  |
| 6 | HF* | g | -111.294 |  |  |
| 13 | HF* | g | -112.97 |  |  |
| 23 | HF* | g | -113 |  |  |
| ^[[26]](#endnote-27)^ | Methanol | aq | -175.31 | -245.93 |  |
| 4 | Methanol | aq | -175.234 | -245.977 |  |
| 20 | Methanol | aq | -175.937 | -246.312 |  |
| 13 | Methanol | aq | -175.39 |  |  |

* Listed in reference as formaldehyde. Revealed in this work as hydrolyzed formaldehyde.

# Glossary of abbreviations and nomenclature

FAld Anhydrous formaldehyde

HF Hydrolyzed formaldehyde

MG Methylene glycol

MeOH Methanol

NAD Oxidized nicotinamide adenine dinucleotide

NADH Reduced nicotinamide adenine dinucleotide

aq Aqueous phase

liq Liquid phase

g Gas phase

c Concentration

Keq Equilibrium constant

R Universal gas constant

T Temperature in Kelvins

F Faraday’s constant

H^+^ Hydrogen ion

e^-^ Electron

$\Delta_{\text{r}}G$ Gibbs energy of reaction

$\varepsilon$ Reduction potential

$\Delta_{\text{f}}G$ Gibbs energy of formation

$\Delta_{\text{f}}H$ Enthalpy of formation

$\Delta_{\text{f}}S$ Entropy of formation

$C_{\text{p}}$ Heat capacity at constant pressure

$\nu_{k}$ Stoichiometric coefficient for species k

$G$ Gibbs energy

$G'$ Transformed Gibbs energy

$G_{\text{w}}$ Gibbs energy of water

$n_{\text{w}}$ Number of water entities in a molecule

# References

1. [] F. Lee Rodkey, “The Effect of Temperature on the Oxidation-Reduction Potential of the Diphosphopyridine Nucleotide System”, *J. Biol. Chem.* **234** (1959) 188-190. [↑](#endnote-ref-2)
2. [] K. Burton and T. H. Wilson, “The free-energy changes for the reduction of diphosphopyridine nucleotide and the dehydrogenation of L-malate and L-glycerol 1-phosphate”, *Biochem J.* **54** (1) (1953) 86-94. DOI: 10.1042/bj0540086. [↑](#endnote-ref-3)
3. [] HSC Chemistry 6. Outotec Research Oy, Pori, Finland, 2006. [↑](#endnote-ref-4)
4. [] Donald D. Wagman, William H. Evans, Vivian B. Parker, Richard H. Schumm, Iva Halow, Sylvia M. Bailey, Kenneth L. Churney, and Ralph L. Nuttall "The NBS tables of chemical thermodynamic properties. Selected values for inorganic and C1 and C2 organic substances in SI units", *J. Phys. Chem. Ref. Data* **11** (1982) Suppl. 2. [↑](#endnote-ref-5)
5. [] F. Walker, “The State of Formaldehyde in Aqueous Solutions”, *J. Phys. Chem.* **35** (4) (1931) 1104-1113, DOI: 10.1021/j150322a015 [↑](#endnote-ref-6)
6. [] J. F. Walker (1944) Formaldehyde, by J. Frederic Walker, Reinhold publishing corporation, New York (1944) 397 pages. [↑](#endnote-ref-7)
7. [] P. G. Russell, N. Kovac, S. Srinivasan, and M. Steinberg, “The Electrochemical Reduction or Carbon Dioxide, Formic Acid, and Formaldehyde”, *J. Electrochem. Soc.: Electrochemical science and technology* **124** (9) (1977) 1329-1338. [↑](#endnote-ref-8)
8. [] J. G. M. Winkelman, O. K. Voorwinde, M. Ottens, A. A. C. M. Beenackers, L. P. B. M. Janssen, “Kinetics and chemical equilibrium of the hydration of formaldehyde”, *Chemical Engineering Science* **57** (19) (2002) 4067-4076, DOI: 10.1016/S0009-2509(02)00358-5. [↑](#endnote-ref-9)
9. [] R. Golden and M. Valentini, “Formaldehyde and methylene glycol equivalence: Critical assessment of chemical and toxicological aspects”, *Regulatory Toxicology and Pharmacology* **69** (2014) 178–186, DOI: 10.1016/j.yrtph.2014.03.007. [↑](#endnote-ref-10)
10. [] da Silva G, Bozzelli JW, Sebbar N, and Bockhorn H, "Thermodynamic and ab initio analysis of the controversial enthalpy of formation of formaldehyde", *Chemphyschem.* **7** (5) (2006) 1119-1126, DOI: 10.1002/cphc.200500667. [↑](#endnote-ref-11)
11. [] B. Ruscic and D. H. Bross, “Active Thermochemical Tables (ATcT) values based on ver. 1.122 of the Thermochemical Network”, https://atct.anl.gov/Thermochemical%20Data/version%201.122/species/?species_number=1008, retrieved November 20, 2019. [↑](#endnote-ref-12)
12. [] F. Walker, “Some Properties of Anhydrous Formaldehyde”, *J. Am. Chem. Soc.* **55** (1933) 2821-2826. [↑](#endnote-ref-13)
13. [] R. K. Thauer, K. Jungermann, and K. Decker, “Energy Conservation in Chemotrophic Anaerobic Bacteria”, *Bacteriological Reviews* **41** (1) (1977), 100-180. [↑](#endnote-ref-14)
14. [] H. Y. Afeefy, J. F. Liebman, and S. E. Stein, "Neutral Thermochemical Data" in NIST Chemistry WebBook, NIST Standard Reference Database Number 69, Eds. P. J. Linström and W. G. Mallard, National Institute of Standards and Technology, Gaithersburg MD, 20899, DOI: 10.18434/T4D303, http://webbook.nist.gov/cgi/cbook.cgi?ID=C50000&Mask=1F, (retrieved November 20, 2019). [↑](#endnote-ref-15)
15. [] R. A. Kiper, “Properties of substance: methanal”, http://chemister.ru/Database/properties-en.php?dbid=1&id=168, (retrieved November 20, 2019). [↑](#endnote-ref-16)
16. [] NIST-JANAF Thermochemical Tables, NIST Standard Reference Database 13, (1998), National Institute of Standards and Technology, Gaithersburg, MD, USA, DOI: 10.18434/T42S31. [↑](#endnote-ref-17)
17. [] J. B. Burkholder, S. P. Sander, J. Abbatt, J. R. Barker, R. E. Huie, C. E. Kolb, M. J. Kurylo, V. L. Orkin, D. M. Wilmouth, and P. H. Wine "Chemical Kinetics and Photochemical Data for Use in Atmospheric Studies, Evaluation No. 18," JPL Publication 15-10, Jet Propulsion Laboratory, Pasadena, 2015 http://jpldataeval.jpl.nasa.gov. [↑](#endnote-ref-18)
18. [] Winkelman, J. G. M. (2003). “Absorption of formaldehyde in water” Groningen: s.n. Chemical Engineering Science 57 (2002) 4067 – 4076 [↑](#endnote-ref-19)
19. [] G. da Silva, J. W. Bozzelli, N. Sebbar, and H. Bockhorn, “Thermodynamic and Ab Initio Analysis of the Controversial Enthalpy of Formation of Formaldehyde”, *ChemPhysChem* **7** (2006) 1119-1126, DOI: 10.1002/cphc.200500667. [↑](#endnote-ref-20)
20. [] OrganoBioGeoTherm, Laboratory of Theoretical Geochemistry and Biogeochemistry, Department of Geology and Geophysics, University of California, (2006), Berkeley, USA. [↑](#endnote-ref-21)
21. [] Shock *et al.*, 1998, “slop98.dat”, DOI: 10.5281/zenodo.2630820. [↑](#endnote-ref-22)
22. [] P. Atkins and J. de Paula, “Atkin’s Physical Chemistry 7^th^ edition”, Oxford University Press (2006) Oxford, United Kingdom. [↑](#endnote-ref-23)
23. [] T. Stretton, ”Organic Compounds: Physical and Thermochemical Data”, (2004), http://www2.ucdsb.on.ca/tiss/stretton/database/organic_thermo.htm, retrieved November 20, 2019. [↑](#endnote-ref-24)
24. [] B. Ruscic and D. H. Bross, “Active Thermochemical Tables (ATcT) values based on ver. 1.122 of the Thermochemical Network”, https://atct.anl.gov/Thermochemical%20Data/version%201.122/species/?species_number=37, retrieved November 20, 2019. [↑](#endnote-ref-25)
25. [] G. Czakó, B. Nagy, G. Tasi, Á, Somogyi, J. Šimunek, J. Noga, B. J. Braams, J. M. Bowman, Császár, and G. Attila, “Proton affinity and enthalpy of formation of formaldehyde”, *Int. J. Quantum Chem.* **109** (11) (2009) 2393-2409, DOI: 10.1002/qua.22009. [↑](#endnote-ref-26)
26. [] R. A. Alberty, “Biochemical Thermodynamics: Applications of Mathematica”, John Wiley & Sons, Inc. (2006) Hoboken, New Jersey, USA. [↑](#endnote-ref-27)
