# Supplementary material for: Evaluation of synthetic formaldehyde and methanol assimilation pathways in Yarrowia lipolytica
Source: Fungal Biol Biotechnol. 2019 Dec 17;6:27. doi: 10.1186/s40694-019-0090-9 (PMC6918578; doi:10.1186/s40694-019-0090-9)
Supplement: Supplementary file 2 — Additional file 2: Document S2. Modification to genome scale model iNL895. [file 40694_2019_90_MOESM2_ESM.docx]

Description of specific changes, reaction additions and reaction direction constraint changes, introduced to *Yarrowia lipolytica* genome-scale model iNL895:

| **rxn_id** | **reaction** | **modified lb** | **iNL895 lb** | **modified ub** | **iNL895 ub** |
| --- | --- | --- | --- | --- | --- |
| r_010_xxx | glycinamide ribonucleotide transformylase | 0 | -Inf | Inf | Inf |
| r_39_exchange | bicarbonate exchange | 0 | -Inf | Inf | Inf |
| r_0125 | acetyl-CoA hydrolase | -Inf | -Inf | 0 | Inf |
| r_0127 | acetyl-CoA synthatase [cytosol] | -Inf | -Inf | 0 | Inf |
| r_0128 | acetyl-CoA synthatase [mitochondrion] | -Inf | -Inf | 0 | Inf |
| r_0129 | acetyl-CoA synthatase [nuclear] | -Inf | -Inf | 0 | Inf |
| r_019_xxx | phosphoribosylpyrophosphate synthetase | 0 | -Inf | Inf | Inf |
| r_0350 | D-arabinono-1,4-lactone oxidase | 0 | -Inf | Inf | Inf |
| r_0371 | Diacylglycerol pyrophosphate phosphatase |  |  |  |  |
| r_0455 -r_0467 | fatty-acyl-ACP synthase | 0 | -Inf | 0 | Inf |
| r_0477 | ubiquinone-coupled formate dehydrogenase | 0 | -Inf | 0 | Inf |
| r_0743 | NADH kinase | 0 | -Inf | Inf | Inf |
| r_0744 | NADH kinase mitochondrial | 0 | -Inf | Inf | Inf |
| r_0746 | NADP phosphatase | 0 | -Inf | Inf | Inf |
| r_0747 | NADP phosphatase | 0 | -Inf | Inf | Inf |
| r_128_exchange | oxygen exchange | 0 | 0 | Inf | 10 |
| r_2000 | decane exchange | -Inf | -Inf | 0 | Inf |
| r_2003 | hexadecane exchange | -Inf | -Inf | 0 | Inf |
| r_2009 | tributyrin exchange | -Inf | -Inf | 0 | Inf |

**rxn_id comment on the modification of bound**

r_010_xxx Would generate ATP if allowed to go to backward direction

r_39_exchange Removed mitochondrial fatty-acyl-ACP synthase reactions for longer than C8 fatty acids as in the yeast 7.6 model

r_0125 Shouldn't be able to make acetyl-CoA from acetate without energy as in this reaction. Directionality restricted as in the yeast 7.6 model

r_0127 All acetyl-CoA to be made via the ATP citrate lyase

r_0128 All acetyl-CoA to be made via the ATP citrate lyase

r_0129 All acetyl-CoA to be made via the ATP citrate lyase

r_019_xxx Shouldn’t be able to generate energy by breaking PRPP. Directionality restricted as in the yeast 7.6 model

r_0350 Reaction is favoured towards D-arabinono-1,4-lactone

r_0371 Diacylglycerol pyrophosphate phosphatase moved from cytocol to ER as in the yeast 7.6 model: r_0371 was removed and a corresponding reaction added to ER.

Diacylglycerol acetyltransferase in endoplasmic reticulum and triglyceride transport from ER to cytosol were added as in yeast 7.6.

r_0477 Removed as in the yeast 7.6 model

r_0743 Directionality restricted as in the yeast 7.6 model

r_0744 Directionality restricted as in the yeast 7.6 model

r_0746 Directionality restricted as in the yeast 7.6 model

r_0747 Directionality restricted as in the yeast 7.6 model

r_128_exchange Free oxygen (O_2_) uptake allowed

r_2000 Only methanol allowed as a carbon source

r_2003 Only methanol allowed as a carbon source

r_2009 Only methanol allowed as a carbon source

**Methanol-related reactions added to the model:**

**Methanol dehydrogenase (EC 1.1.1.244)**

methanol + NAD <=> formaldehyde + NADH

**Methanol and formaldehyde export and transport reactions**

<=> methanol_external

methanol_cytosolic <=> methanol_external

<=> formaldehyde_external

formaldehyde_cytosolic <=> formaldehyde_external

**XuMP pathway**

**formaldehyde transketolase (EC 2.2.1.3)**

formaldehyde + xylulose-5p <=> glycerone + glyceraldehyde-3p

**RuMP pathway**

**3-hexulose-6-phosphate synthase (EC 4.1.2.43)**

formaldehyde + ribulose-5p <=> 3-hexulose-6-phosphate

**6-phospho-3-hexuloisomerase (EC 5.3.1.27)**

3-hexulose-6-phosphate <=> fructose-6-phosphate

**Serine cycle**

**formaldehyde to methylenetetrahydrofolate**

formaldehyde + tetrahydrofolate <=> 5,10 -methylenetetrahydrofolate

**serine-glyoxylate aminotransferase**

glyoxylate + serine <=> glycine + hydroxypyruvate

**hydroxypyruvate reductase**

hydroxypyruvate + NADP <=> glycerate + NADPH

**glycerate kinase**

glycerate + ATP <=> 3-phosphoglycerate + ADP

**phosphoenolpyruvate carboxylase**

phosphate + oxaloacetate = phosphoenolpyruvate + HCO3-

**malate-Coa ligase**

malate + ATP + coenzyme A <=> Malyl-CoA + phosphate + ADP

**malyl-Coa lyase**

malyl-CoA <=> acetyl-CoA + glyoxylate
