# Supplementary material for: Evaluation of synthetic formaldehyde and methanol assimilation pathways in Yarrowia lipolytica
Source: Fungal Biol Biotechnol. 2019 Dec 17;6:27. doi: 10.1186/s40694-019-0090-9 (PMC6918578; doi:10.1186/s40694-019-0090-9)

Extracellular metabolites analysed from bioreactor cultivations

Bioreactor cultivations were performed in 15-unit bioreactor (Medicel, Finland) in the defined minimal medium described by Verdyun *et al.* [40], with 5 g/L glycerol or 10 g/L yeast extract and 32 g/L methanol as carbon source. BDH silicon antifoam was used to prevent foaming of the cultures. The culture conditions were as follows: 200 ml volume, pH 5.0, +30°C and with 0.5 vvm air.

1. Cultivations with 5 g/L glycerol and 32 g/L methanol as a carbon source


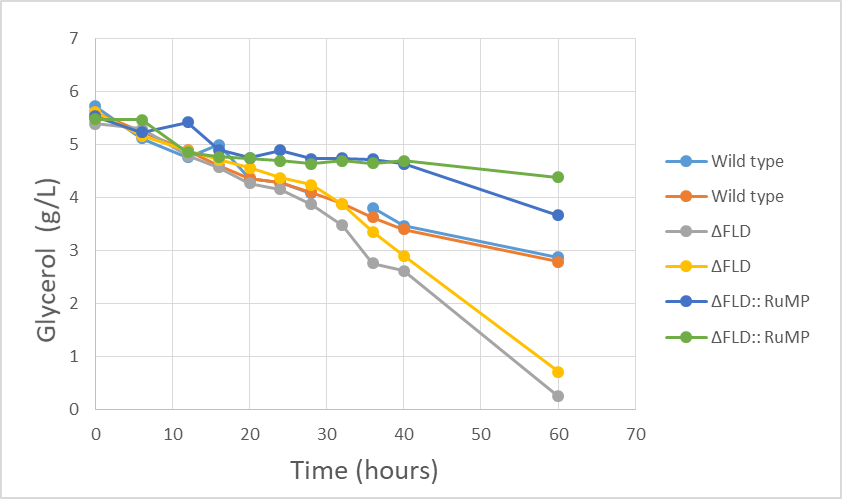


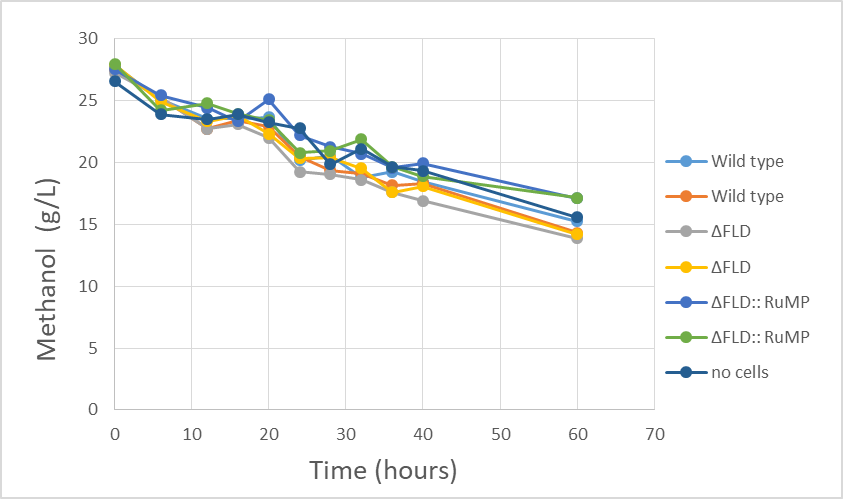


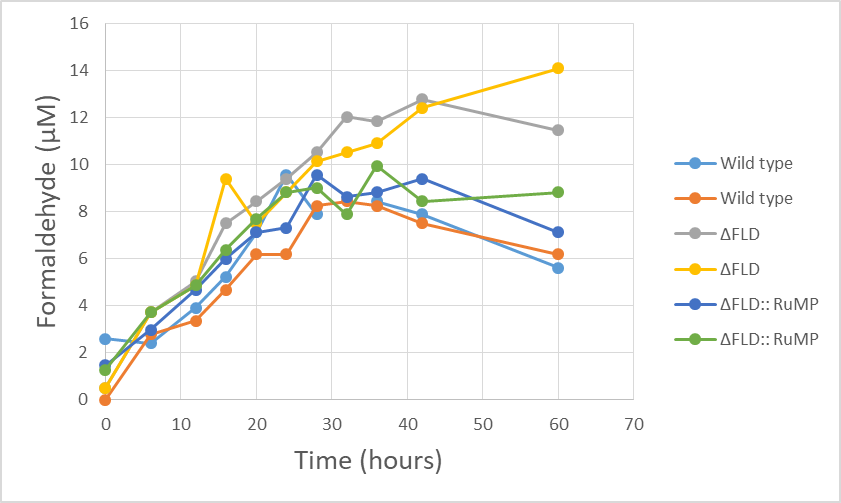


1. Cultivations with 10 g/L glycerol and 32 g/L methanol as a carbon source


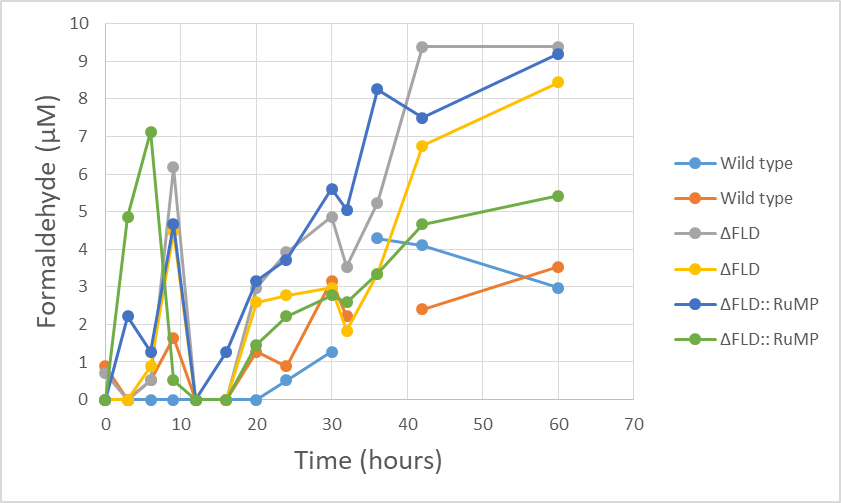

Supplement: Supplementary file 3 — Additional file 3: Document S3. Extracellular metabolites analysed from bioreactor cultivations. [file 40694_2019_90_MOESM3_ESM.docx]
